# Warifteine, an Alkaloid Purified from *Cissampelos sympodialis*, Inhibits Neutrophil Migration *In Vitro* and *In Vivo*

**DOI:** 10.1155/2014/752923

**Published:** 2014-06-05

**Authors:** Thaline F. A. Lima, Juliana D. B. Rocha, Anderson B. Guimarães-Costa, José M. Barbosa-Filho, Débora Decoté-Ricardo, Elvira M. Saraiva, Luciana B. Arruda, Marcia R. Piuvezam, Ligia M. T. Peçanha

**Affiliations:** ^1^Departamento de Imunologia, Instituto de Microbiologia Prof. Paulo de Góes, Universidade Federal do Rio de Janeiro, CCS, Bloco I, Sala I2-062, Ilha do Fundão, 21944-570 Rio de Janeiro, RJ, Brazil; ^2^Departamento de Fisiologia e Patologia, Universidade Federal da Paraíba, 58051-970 João Pessoa, PB, Brazil; ^3^Instituto de Veterinária, Universidade Federal Rural do Rio de Janeiro, 23890-000 Seropédica, RJ, Brazil; ^4^Departamento de Virologia, Instituto de Microbiologia Prof. Paulo de Góes, Universidade Federal do Rio de Janeiro, 21944-570 Rio de Janeiro, RJ, Brazil

## Abstract

*Cissampelos sympodialis* Eichl is a plant from the Northeast and Southeast of Brazil. Its root infusion is popularly used for treatment of inflammatory and allergic diseases. We investigated whether warifteine, its main alkaloid, would have anti-inflammatory effect due to a blockage of neutrophil function. *In vivo* warifteine treatment inhibited casein-induced neutrophil migration to the peritoneal cavity but did not inhibit neutrophil mobilization from the bone marrow. Analysis of the direct effect of warifteine upon neutrophil adherence and migration *in vitro* demonstrated that the alkaloid decreased cell adhesion to P and E-selectin-transfected cells. In addition, fLMP-induced neutrophil migration in a transwell system was blocked by warifteine; this effect was mimicked by cAMP mimetic/inducing substances, and warifteine increased intracellular cAMP levels in neutrophils. The production of DNA extracellular traps (NETs) was also blocked by warifteine but there was no alteration on PMA-induced oxidative burst or LPS-stimulated TNF**α** secretion. Taken together, our data indicate that the alkaloid warifteine is a potent anti-inflammatory substance and that it has an effect on neutrophil migration through a decrease in both cell adhesion and migration.

## 1. Introduction

*Cissampelos sympodialis* is a plant found in the Northeast and Southeast regions of Brazil [1] and its extract is popularly used in the treatment of both inflammatory and allergic diseases [2]. Studies using the aqueous fraction of the ethanolic extract obtained from its leaves (AFL) have confirmed its immunomodulatory effects. AFL was shown to decrease T cell, B cell, and macrophage function* in vitro* [3–5].* In vivo* studies showed that AFL treatment decreased IgE levels in ovalbumin sensitized animals [6, 7] and inhibited carrageenan-induced edema [8]. These findings suggest that components present in the AFL may be important for the anti-inflammatory and antiallergic effects of* C. sympodialis*.

Warifteine, a bisbenzylisoquinoline alkaloid, is the major alkaloid purified from both root and leaves of* C. sympodialis* [2, 9]. Warifteine was described to induce spasmolytic activity on tracheal and cardiac smooth muscle and to inhibit calcium channels and to modify the intracellular Ca^2+^ stores [10, 11]. Also, this alkaloid had immunomodulatory effects since it reduced T cell response and IgE production in an ovalbumin-induced allergy model, increased the levels of regulatory T cells in a food allergy model [12, 13], and decreased B cell proliferation and immunoglobulin production [14]. This late effect was mediated through an increase in intracellular cAMP levels [14].

Neutrophils are key cells in the inflammatory response which migrate to inflamed tissue towards the higher concentration of locally produced chemotactic substances [16]. The migratory response is a multistep process that is initiated by a selectin-dependent neutrophil rolling on endothelial cells [17]. Subsequently, an LFA-1-mediated arrest occurs and this is followed by lateral motility of leukocytes on the surface of endothelial cells which is mediated by the Mac-1 integrin and this is followed by neutrophil transendothelial migration [18]. Blockage of any of the steps in the described processes will decrease the magnitude of the inflammatory response and induce severe infection-associated disease [19, 20]. Neutrophils will participate in the local response through the production of several antimicrobial substances like the induction of oxidative burst [21]. Also, neutrophils were shown to kill microorganisms through the production of neutrophil extracellular traps (NETs) which are formed by the chromatin associated to granular and cytoplasm components [22, 23]. NETs can be induced by IL-8, LPS, fMLP, PMA, bacteria, fungi, and the protozoan* Leishmania *as well as by HIV [24–27]. Moreover, NETs were recently identified in human atopic asthmatic airways* in vivo* [28, 29]. Despite NETs' reported microbicidal function, exaggerated NET formation may be also associated with pathological conditions like autoimmune and inflammatory disorders [30, 31].

Several models have been employed for measuring neutrophil mobilization* in vivo*. Casein injection was shown to induce a massive increase in neutrophil numbers in the peritoneal cavity [32, 33]. The neutrophil levels reach an average content of 75%. Those cells are only partially activated, since they still respond to inflammatory stimuli [34, 35].

Given that warifteine has been described as a potential anti-inflammatory compound, in the present study we investigated whether the anti-inflammatory effect of warifteine would be mediated through an inhibition of neutrophil mobilization and response* in vitro *and* in vivo*. We employed here the model of neutrophil migration to the peritoneal cavity after injection of casein.

## 2. Materials and Methods

### 2.1. Reagents

**Figure 1:**
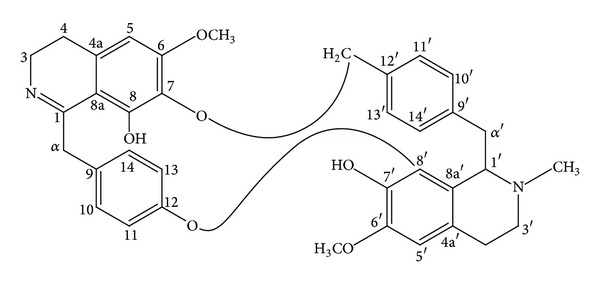
Structure of warifteine. The structure is based on data previously published [7, 15].

DMEN tissue culture medium, fetal calf serum, and gentamicin (used at 50 *μ*g/mL) were obtained from GIBCO (Invitrogen Corporation, Carlsbad, CA, USA). This medium was supplemented with HEPES, sodium bicarbonate, l-glutamine, and *β*-mercaptoethanol (obtained from Sigma-Aldrich, St. Louis, MO, USA). Warifteine was purified from roots and leaves of plants grown in the botanical garden of Departamento Fisiologia e Patologia from Federal University of Paraiba. A voucher specimen (Agra-1456) was deposited at Herbarium Lauro Pires Xavier from the Federal University of Paraiba. Hydroalcoholic material from dried roots and leaves was extracted with 80% ethanol. This step was followed by several extractions with chloroform and a final purification by TLC [12, 36]. Detailed information on the purification and structure of the warifteine was previously published [2, 15] and its structure is shown in Figure 1. The warifteine used throughout was in its crystal form and had a purity of 100%. Warifteine was endotoxin free, as determined by NMR and mass spectroscopy. The warifteine solutions were prepared by adding 50 *μ*L of HCl 1 N to 1 mg of the crystal followed by the addition of 800 *μ*L of distilled water. The pH was adjusted to 7.0 with NaOH 1 N. The dilutions were made in PBS [12]. Under these conditions the alkaloid showed 100% solubility. Lipopolysaccharide (LPS) (extracted from* E. coli* 0111:B4), casein, phorbol myristate acetate (PMA), and N-formyl-methionine-leucine-phenylalanine (fMLP) were purchased from Sigma-Aldrich (St. Louis, MO, USA).

### 2.2. Animals and Treatments

Male and female BALB/c mice with age ranging from 6 to 8 weeks were obtained from the animal facility of the Instituto de Microbiologia from Federal University of Rio de Janeiro. The animals were bred and housed according to institutional policies for animal care and usage. All experimental protocols were approved by the Ethical Committee from the Center of Health Sciences from Federal University of Rio de Janeiro (protocol no. IMIPPG 012) and adhered strictly to the guidelines of the Brazilian Law no. 11.794 from 2008 which established the rules for use and care of laboratory animals.

For* in vivo* studies, mice were injected (i.p.) with a 9% solution of casein diluted in PBS. Warifteine-treated animals were injected i.p. with the alkaloid (10 *μ*g/mice) 1 h before casein inoculation. The doses used were established in previous dose-response studies and were in accordance with the doses described in previous reports [7, 37]. For bone marrow analysis, mice were kept untreated or were injected i.p. with casein, warifteine, or both substances. Cells were obtained 3 h later from tibia and femur. Samples containing 2 × 10^5^ cells were put into microscopy slides. A stained cytospin preparation was set up. Differential cell counting was performed by analyzing 300 cells per slide. For blood cell analysis, samples were obtained from mice treated as described above. Three hours after treatment with casein, a drop of blood was obtained from the lateral tail vein and a blood smear was prepared. The samples were stained with Giemsa dye. Differential cell counting was performed after analyzing a total of 1000 cells per slide. Neutrophil percentage in peritoneal exudates was determined by optical microscopy. Peritoneal exudates were obtained after warifteine treatment followed by a 3 h treatment with casein. Samples (2 × 10^5^ cells) were applied to microscopy slides and they were submitted to cytospin. The slides were then stained with May-Grunwald Giemsa. Percentage of neutrophils in the samples was obtained by differential cell count. Around 300 cells were counted in each slide. The PMN cell population in peritoneal exudates was also identified by flow cytometry using a FACSCalibur (BD Biosciences, San Jose, CA, USA). Forward scatter (FSC) by side scatter (SSC) profile was determined and the number of neutrophils was counted in the PMN gate. Gr-1 expression in the gated population was also employed to confirm these cells were neutrophils. A total of 10,000 cells were analyzed by flow cytometry. Data were analyzed using the Cell Quest Software.

### 2.3. Neutrophil Enrichment from Peritoneal Exudates for* In Vitro* Studies

*In vitro* studies were performed using neutrophil-rich exudates obtained from casein-treated mice as described above. The cells were obtained 3 h after casein injection and the material was analyzed for neutrophil percentage in stained cytospin preparations before use. Treatment was performed using the protocol described by Luo and Dorf [37]. This protocol provides a neutrophil enriched cell preparation with up to 75% neutrophils as determined by flow cytometry. This neutrophil-rich cell preparation will be called neutrophils throughout the paper for simplicity.

### 2.4. Cell Viability Studies

Propidium iodide labeling [14]: neutrophils were obtained from the peritoneal cavity of mice 3 h after i.p. injection of casein. These cells were cultured for 90 min in either the presence or absence of PMA (1 nM) and warifteine (added at various concentrations 15 min before PMA addition). This was followed by staining with a FITC-labeled anti-Gr1 antibody (clone RB6-8C5, BD Pharmingen). Afterwards, propidium iodide (10 *μ*L at 2.5 *μ*g/mL in saline) was added to the samples that were incubated for 30 min and subsequently analyzed. The samples were analyzed for propidium iodide labeling by flow cytometry.

XTT cell viability assay [38]: neutrophils were obtained from the peritoneal cavity of casein-treated mice. These cells were cultured for 90 min in either the presence or absence of the following reagents: LPS (100 ng/mL), PMA (1 nM), and warifteine (added at a range of concentrations 15 min before addition of either PMA or LPS). After incubation, the cells were treated for 1 to 4 h with 50 *μ*L of XTT solution (used at 20 *μ*g/mL) at 37°C under a 7% CO_2_ atmosphere. Triton X-100-treated cultures were used as positive (dead cells) controls. Cell viability was visualized by the development of a colored orange derivative by viable cells. Plates were then read using a microplate reader for determining A_490_.

### 2.5. Reactive Oxygen Species (ROS) Measurement

A suspension of neutrophils obtained from the peritoneal cavity of casein-injected mice was treated with PMA* in vitro* (1 nM). Some cultures also received different doses of warifteine, which was added 15 min before PMA treatment. The samples were incubated for 1 h at 37°C and were then incubated with the CM-H_2_DCFDA probe (2.5 *μ*M, Molecular Probes, Invitrogen Corporation, Carlsbad, CA, USA) for 30 min in the dark at 37°C in an atmosphere containing 7% CO_2_. The samples were then analyzed by flow cytometry for measuring ROS production.

### 2.6. Cell Binding Assay

CHO cells and CHO cells transfected with the genes for E-selectin (CHOE) or P-selectin (CHOP) [39] were added to 24-well plates containing a coverslip and the cultures were incubated overnight for establishing a cell monolayer. Cell suspensions were prepared in DMEM culture medium supplemented with 10% FCS. The following day a neutrophil-rich cell suspension was obtained from the peritoneal cavity of mice pretreated with casein. Some cultures were incubated with different doses of warifteine for 15 min. After incubation, the neutrophil samples were added to cultures containing a monolayer of CHO, CHOE, or CHOP cells. The coculture was carried out for 90 min. Afterwards, the plates were washed with PBS to remove nonadherent cells and the coverslips were stained with Giemsa, mounted on slides, and counted by light microscopy. Around 500 CHO cells were counted and the number of neutrophils that adhered per cell was determined.

### 2.7. *In Vitro* Transwell Cell Migration Assay

Cell chemotaxis was determined using a transwell system (Corning, NY, USA). Transwell inserts with a 5 *μ*m polycarbonate membrane were used. Neutrophils (1.5 × 10^6^) were left untreated or were incubated with different doses of warifteine for 15 min at 37°C in a 7% CO_2_ atmosphere. The cell samples were added into transwell inserts placed in a 24-well plate containing fMLP (20 nM) in the outside compartment. The migration system was incubated for 1 h at 37°C in a 7% CO_2_ atmosphere. The number of cells in the fMLP compartment was determined by direct cell counting using a hemocytometer. The chemotaxis index for each sample was calculated by the ratio of the cell number in the outside compartment in cultures with chemotactic reagents and the cell number in medium only cultures (random spontaneous cell migration). DbAMPc or forskolin treatment was performed as follows: cell samples of 1.5 × 10^6^ neutrophils were incubated for 15 min with warifteine (2 *μ*M), DbAMPc (2 *μ*M), or forskolin (10 *μ*M). Cultures were set up as described above. Pretreated cell samples were added to transwell inserts and fMLP was added in the outside compartment as described above.

### 2.8. Measurement of Neutrophil Extracellular DNA Traps (NETs)

Extracellular DNA was measured as previously described [25]. Neutrophils (10^6^) were incubated with several doses of warifteine for 15 min. The cells were then stimulated with fMLP (20 nM). The cultures supernatants were obtained 1 h later and treated with the restriction enzymes ECOR1 and HINDIII (20 units/mL) and incubated for an additional 2 h period. The supernatants were harvested and the DNA content was quantified using the dsDNA Picogreen kit (Invitrogen Corporation, Carlsbad, CA, USA). NET evaluation by microscopy analysis was performed as follows: neutrophils (2 × 10^5^) were transferred to round 13 mm coverslips and incubated for 15 min with different doses of warifteine. Afterwards the cells were stimulated with fMLP as shown above for 45 min. The coverslips were then fixed with 4% paraformaldehyde and stained with DAPI. The samples were analyzed by phase contrast and epifluorescence microscopy using a Zeiss Axioplan microscope.

### 2.9. Measurement of Intracellular cAMP

Cyclic AMP was quantified using Gilman's competitive binding assay [40] modified as previously described [41]. Briefly, neutrophils were incubated for 10 min at 37°C in RPMI medium (pH 7.2) containing 0.5 mM isobutylmethylxanthine and 100 M ascorbic acid and incubated for 1 h with either warifteine (2 *μ*M) or forskolin (10 *μ*M—that raises levels of cAMP and was used as a positive control). The reaction was stopped by the addition of trichloroacetic acid. The samples were centrifugated and the supernatant was individually passed through an ion-exchange resin column (Dowex 50) to remove trichloroacetic acid and other nucleotides. The samples obtained were then used in a competition assay with the regulatory subunit of PKA with the addition of a fixed, trace amount of [^3^H]cAMP.

### 2.10. Statistical Analysis

The results are shown as arithmetic means ± SD. Student's *t*-test for independent samples was performed using the PrismGraphPad 4 software. Level of significance was set at *P* ≤ 0.05.

## 3. Results 

### 3.1. Treatment with Warifteine Decreased Cell Migration* In Vivo*

**Figure 2:**
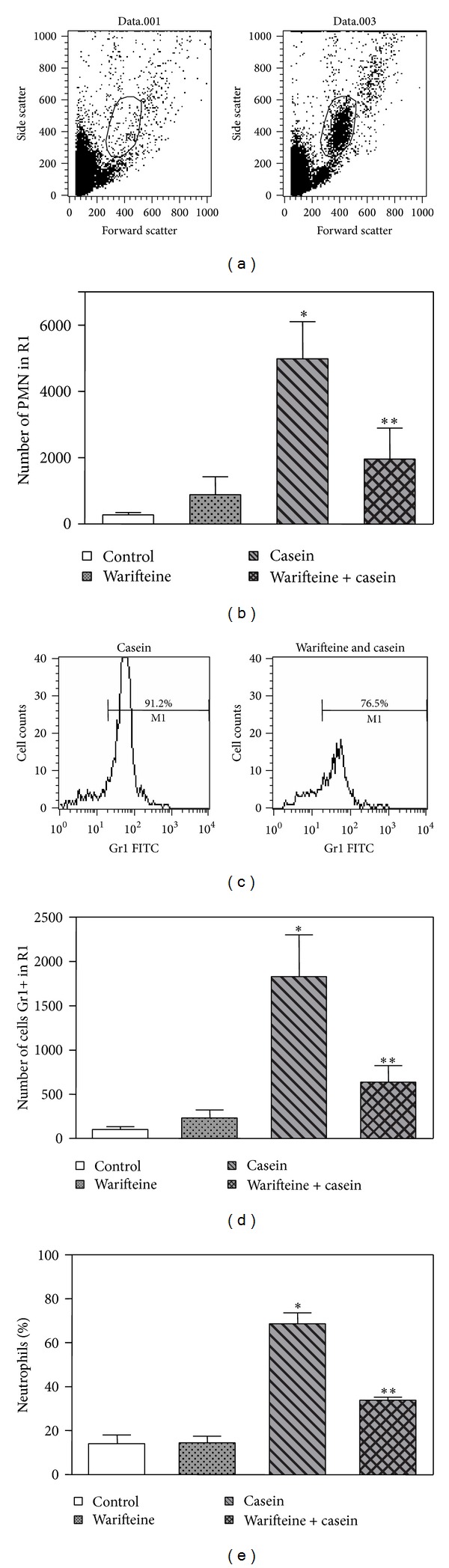
Neutrophil number in the peritoneal cavity of mice treated with casein and warifteine. (a, b) Cell number evaluation by flow cytometry. Peritoneal cells were obtained from mice after treatment with warifteine and casein. The cells were prepared for analysis by flow cytometry. (a) Side scatter and forward scatter profiles of normal (left) and casein-treated (right) animals to indicate the R1 gate. (b) Total PMN cell number in the R1 gate (as defined in (a)). The FSC versus SSC plots are representative of three independent experiments. The mean value of neutrophil numbers in R1 gates from three independent experiments is shown (*n* = 3 animals per group/experiment). *P ≤ 0.05 as compared to untreated controls and **P ≤ 0.05 as compared to casein-treated animals. (c) Analysis of the percentage of Gr1+ cells in the peritoneal cavity fluid from animals treated with casein and warifteine. Peritoneal fluid was obtained from warifteine and casein-treated animals. The samples were incubated with an anti-Gr1-FITC labeled antibody. Histograms show the Gr1-expressing cells in the R1 gate. The value shown in M1 indicates the percentage of Gr1+ cells. Data shown in the histogram plots are representative of two independent experiments. (d) Quantification of the number of Gr1+ cells in the R1 gate. Data shown indicate the total number of GR1+ cells from experiments shown in (a) and are the mean value from two experiments (*n* = 4 animals per group/experiment): *P ≤ 0.05 in comparison with control animals and **P ≤ 0.05 in relation to animals treated with casein alone. (e) Percentage of neutrophils in peritoneal exudate obtained from mice treated with casein and warifteine. Peritoneal exudates were obtained after a treatment with warifteine and casein and were submitted to cytospin followed by May-Grunwald Giemsa staining. Data in (e) show the percentage of neutrophils in the samples obtained by differential cell count. Data shown are mean value from six independent experiments (*n* = 3 animals per group/experiment). *P ≤ 0.05 when comparing with untreated controls and **P ≤ 0.05 when comparing data with those from the group treated with casein only.

The initial studies aimed to identify whether warifteine would have an inhibitory effect on neutrophil migration* in vivo*. Mice were injected with casein and warifteine and the effect of the alkaloid in neutrophil migration was investigated. Intraperitoneal casein injection induced, as previously described, an increase in the percentage of neutrophils in the peritoneal fluid (Figure 2(a)). Peritoneal exudates were obtained between 3 and 5 h after casein injection since kinetic studies have shown that neutrophil migration would peak in this time interval (data not shown). The doses of warifteine used* in vivo* were the ones that were previously characterized as able to significantly inhibit allergic inflammation [7, 42] and to induce maximal inhibition of neutrophil migration in preliminary studies (data not shown). In order to thoroughly investigate the effect of warifteine in neutrophil migration* in vivo*, the inhibitory effect of warifteine in casein-induced* in vivo *neutrophil mobilization was analyzed and determined by three approaches: determination of the number of cells in the PMN gate by flow cytometry analysis in plots of forward scatter (FSC) by side scatter (SSC) (Figures 2(a) and 2(b)), calculation of the percentage of Gr-1+ cells in the PMN gate measured by flow cytometry (Figures 2(c) and 2(d)), and, finally, determination of the percentage of neutrophils in cytospin smears (Figure 2(e)).* In vivo* pretreatment with warifteine inhibited the number of neutrophils in the PMN gate of casein-treated animals in around 60% (Figures 2(a) and 2(b)). Warifteine treatment* in vivo* decreased the percentage of GR1+ cells among the cells in the PMN leukocytes gate (from 91.2 to 76.5%, Figure 2(c)). The total number of Gr1+ cells was diminished in 65% (Figure 2(d)). Finally, the percentage of neutrophils in cytospin smears was also decreased in around 45% (Figure 2(e)).

**Figure 3:**
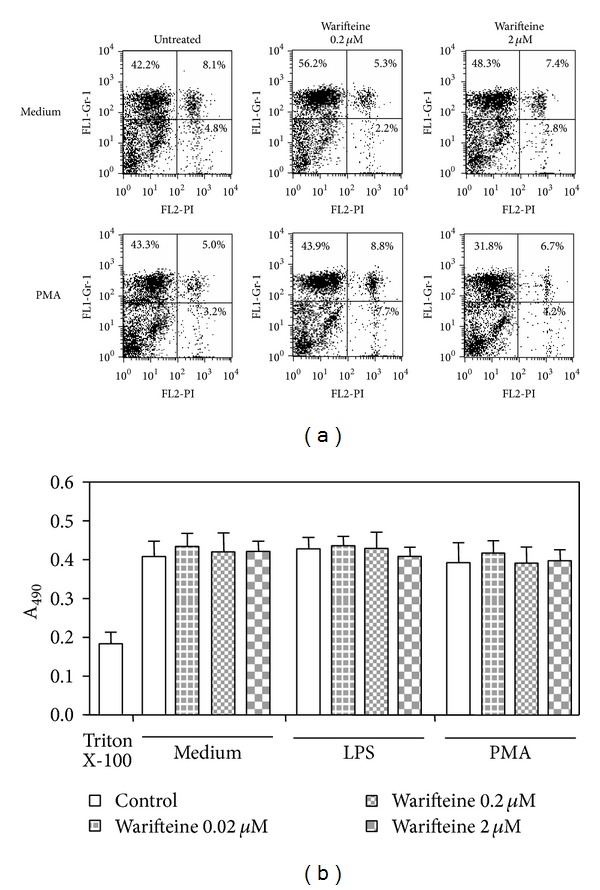
Evaluation of the toxic effect of warifteine on neutrophils. (a) Isolated neutrophil suspensions were incubated with the indicated doses of warifteine and subsequently stimulated with PMA. Cultures were then incubated with FITC-labeled anti-Gr-1 antibody, which was followed by incubation with a solution of propidium iodide. The samples were analyzed for propidium iodide labeling by flow cytometry. The plots indicate the labeling of cells in the PMN gate (as defined in Figure 2) and are representative of two independent experiments. (b) Isolated neutrophil suspensions were treated for 90 min with PMA. Some cultures also received the indicated doses of warifteine. The cells were then incubated with the XTT dye and XTT reduction by viable cells was determined. Triton X-100-treated cultures were used as positive (dead cells) controls. Statistical analysis showed no significant differences between warifteine-treated and untreated groups. Data are representative of three independent experiments.

**Figure 4:**
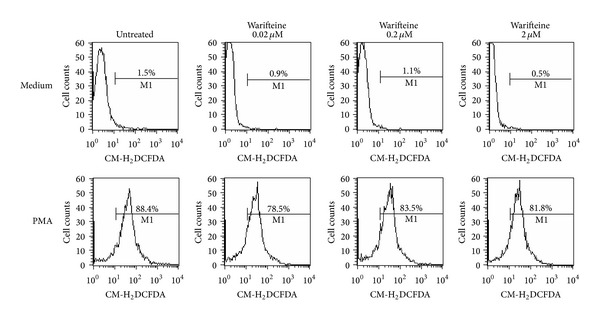
Measurement of reactive oxygen species in cultures stimulated with PMA and treated with warifteine. Neutrophils were incubated in either the presence or absence of PMA. Some cultures were also treated with the indicated doses of warifteine. The samples were then incubated with the probe CM-H_2_DCFDA. The production of ROS was analyzed by flow cytometry. The M1 marker shows the percentage of cells that metabolized the probe. The data are representative of three independent experiments.

**Figure 5:**
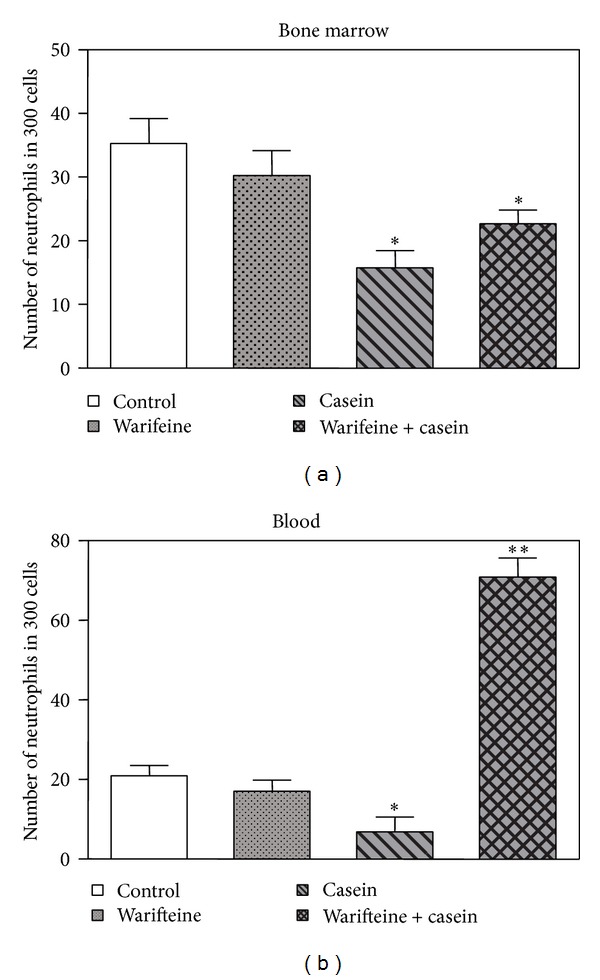
Number of polymorphonuclear leukocytes in the bone marrow and peripheral blood of mice treated with casein and warifteine. Mice were treated with warifteine, casein, or both substances. After a three-hour period past casein treatment, bone marrow (a) and blood samples (b) were obtained. Blood smears and bone marrow cytospins were analyzed by optical microscopy after staining. The graph shown in (a) indicates the mean value from data obtained in two experiments (*n* = 3 animals per group). *P ≤ 0.05 when comparing the group of animals treated with casein with untreated animals (controls). There was no statistical difference between the group treated with casein and the one treated with warifteine plus casein. Data shown in (b) indicate the percentage of blood neutrophils in different groups and are the mean value from three independent experiments (*n* = 3 animals per group/experiment). *P ≤ 0.05 when comparing animals treated with casein with the untreated ones and **P ≤ 0.05 in the comparison of the groups of animals treated with warifteine and casein with the group treated with casein only.

To evaluate whether the decreased cell migration would be due to a direct toxic effect of warifteine, which would decrease total* in vivo* neutrophil numbers, we cultured fresh and PMA-activated neutrophils with the alkaloid and analyzed cell viability by both PI incorporation and XTT metabolization. Warifteine did not affect PI staining of resting or PMA-activated cells (Figure 3(a)). The same pattern of response was observed in LPS-stimulated cultures (data not shown). Cell viability, as measured by the metabolization of the XTT dye, was not modified by warifteine treatment (Figure 3(b)). Warifteine would not induce an overall blockage in neutrophil function since it did not induce a decrease in oxidative burst in neutrophils stimulated by either PMA (Figure 4) or LPS* in vitro* (data not shown). The decrease in neutrophil recruitment to the peritoneal cavity could not also be explained by a decrease in neutrophil mobilization from bone marrow since there was a decline in neutrophil numbers in bone marrow despite the previous treatment with warifteine before casein-induced neutrophil mobilization (Figure 5(a)). Interestingly, unlike what was observed in casein-only treated animals, mice treated with both casein and warifteine showed an accumulation of neutrophils in peripheral blood after warifteine treatment (Figure 5(b)).

### 3.2. Warifteine Reduced Neutrophil Migration through a Decrease in Both Cell Adhesion and Chemotactic Response and Induced an Increase in Intracellular cAMP Levels

**Figure 6:**
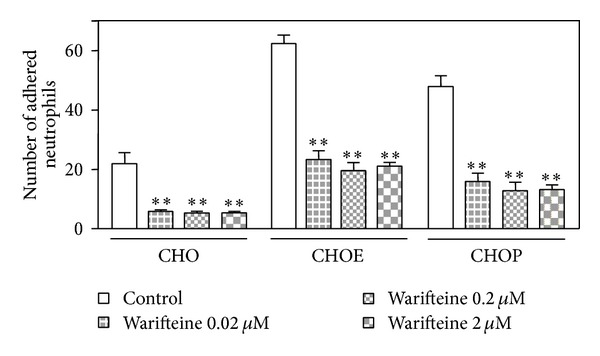
Adhesion of neutrophils to CHO cells and to CHO cells expressing P and E selectin. CHO cells transfected with either P (CHOP) or E selectin (CHOE) genes were used. A cell monolayer was obtained by overnight incubation of normal or transfected CHO cells in coverslips. Peritoneal exudate neutrophils were obtained and treated with warifteine* in vitro*. Those cells were then transferred to the monolayers of CHO cells and incubated. Coverslips were removed, washed with PBS, and Giemsa-stained. They were analyzed by optical microscopy. The number of neutrophils adhered to CHO cells was scored. The graphs shown indicate the mean values from four independent experiments performed in triplicate. **P ≤ 0.05 in the comparison of cultures treated with warifteine (in different concentrations) and cultures treated with medium only without warifteine.

We next investigated the mechanism by which warifteine would decrease neutrophil migration by analyzing its effect on cell adhesion and chemotaxis* in vitro*. We observed that adhesion of unstimulated casein-mobilized neutrophils to CHO cells expressing either P or E selection was blocked by different doses of warifteine, indicating that the compound strongly inhibited the selectin-mediated adhesion of neutrophils (Figure 6).

**Figure 7:**
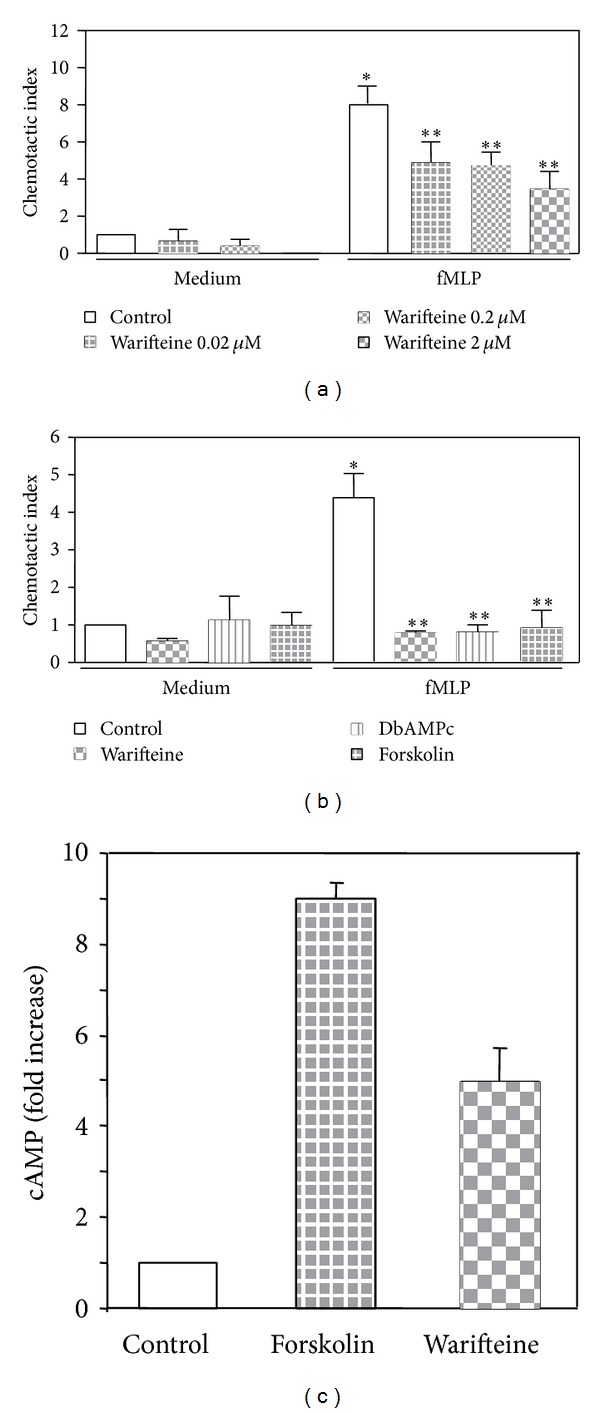
Inhibition of neutrophil migration in the presence of warifteine and induction of increased intracellular cAMP levels. (a) Solutions of chemoattractant (fMLP) were added to a 24-well plate. A neutrophil suspension was obtained and either left untreated or incubated with the indicated doses of warifteine. Theses samples were added to transwell inserts which were placed into the fMLP filled wells. Cell migration was determined by counting cell number in the outside (fMLP) compartment using a hemocytometer. The chemotactic index was calculated for each sample. Data shown indicate the mean value obtained from two independent experiments. *P ≤ 0.05 when comparing untreated and fMLP-stimulated cultures; **P ≤ 0.05 when comparing data from warifteine-treated and untreated cultures. (b) Samples obtained as described in (a) were incubated with warifteine, DbAMPc, or forskolin. Cultures were set up as in (a). Pretreated cell samples were added into a transwell insert placed in fMLP containing wells (outside compartment). The chemotactic index is shown. (c) Neutrophil cultures were incubated with either forskolin or warifteine. Control cultures received no pretreatment. Intracellular cAMP levels were measured by a competitive binding assay. Data indicate the fold increase of cAMP levels compared to untreated controls (set as 1).

Also, the addition of warifteine to transwell chambers blocked the migration of neutrophils induced by the chemoattractants fMLP (Figure 7(a)) and LPS (data not shown). The effect of warifteine on cell chemotaxis was mimicked by the addition of DbcAMP and forskolin (Figure 7(b)) and warifteine treatment induced an increase in intracellular cAMP levels (Figure 7(c)).

### 3.3. Effect of Warifteine on Neutrophil Function

**Figure 8:**
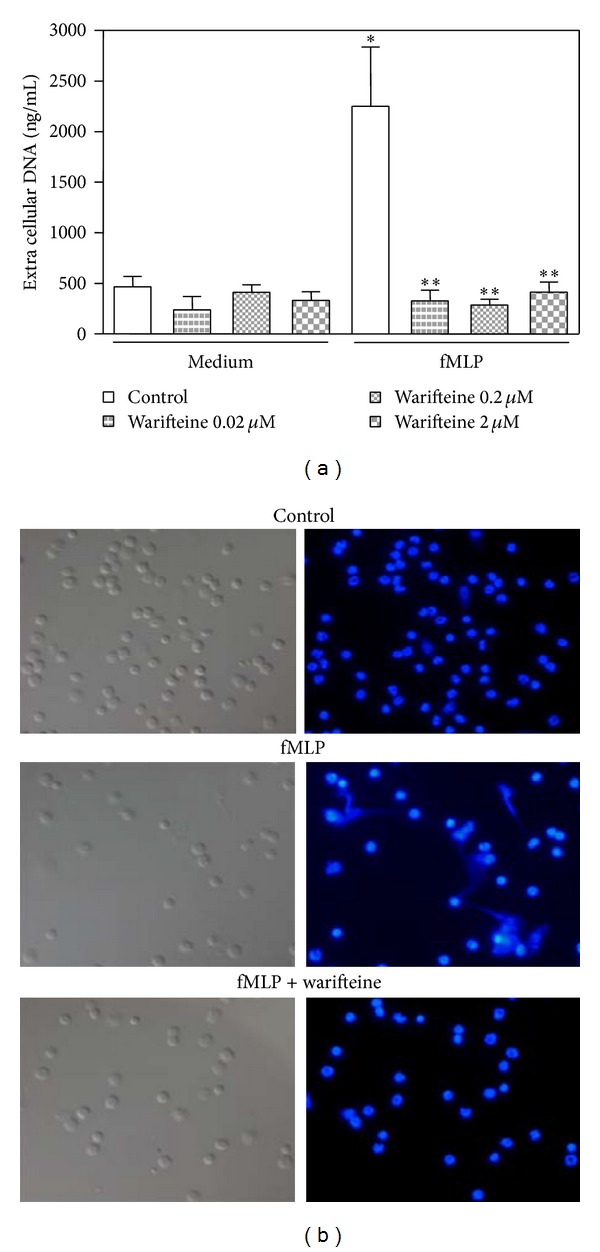
Extracellular DNA traps (NETs) induction in warifteine-treated neutrophil cultures. (a) Neutrophils were preincubated with the indicated doses of warifteine. The cells were then stimulated with fMLP. The supernatants were then harvested and the amount of extracellular DNA was measured. (b) Neutrophils were transferred to coverslips, treated with warifteine (2 *μ*M), and then stimulated with fMLP. The coverslips were fixed and stained with DAPI. This was followed by analysis by phase contrast and fluorescence microscopy. Plots show representative fields recorded in light (right) and dark (left) fields where DAPI labeling was determined. Data are representative of three independent experiments.

We next investigate whether other parameters associated with neutrophil activation would be modified by warifteine. NETs were not induced by the alkaloid, which inhibited NET release induced by fMLP (Figure 8). Warifteine, as shown above, did not decrease induction of oxidative burst in neutrophils stimulated by PMA (Figure 4). Warifteine neither modified the secretion of TNF-*α in vitro* after LPS-induced stimulation (data not shown) nor decreased PMA-induced neutrophil degranulation as measured by flow cytometry, SSC scatter (data not shown).

## 4. Discussion 

Our previous study has shown that the aqueous fraction obtained from the ethanolic extract of roots and leaves of* C. sympodialis* inhibited carrageenan-induced neutrophil migration [8]. The alkaloid warifteine, the main alkaloid isolated from this plant extract, was previously shown to inhibit leukotriene production, which would impart an anti-inflammatory effect to this alkaloid [7, 42]. In the present study we further characterized the anti-inflammatory mechanism of warifteine and described the effect of the alkaloid in neutrophil migration.

A previous study has shown that the IC_50 _for warifteine ranged from 10 to 35 *μ*M when the alkaloid is added to cultures of either fibroblast or hepatic cell lines [9]. These doses are quite higher than the ones we used in the present study. Also, warifteine did not induce an overall blockage in neutrophil function since it did not show any toxic effect on this cell type and we observed that the alkaloid did not block PMA-induced oxidative burst.

Several factors have been shown to induce a rapid blood neutrophilia, like the chemokine MIP-2, which induces both an increase in circulating levels of neutrophils and its accumulation in the peritoneal cavity after thioglycollate injection [43]. The casein-induced neutrophilia was maintained and was even increased after warifteine injection, which indicated that warifteine did not modify neutrophil mobilization from the bone marrow* in vivo*. However, the next step of the process (the migration to the peritoneal cavity), which will depend on cell adhesion, was blocked by warifteine. Our* in vitro* measurement of neutrophil adhesion to selectin-transfected CHO cells directly indicated that selectin-mediated adhesion was blocked by warifteine and this effect can explain the decrease in neutrophil migration to the peritoneal cavity we observed* in vivo*.

Previous studies have characterized alkaloids with anti-inflammatory effect [44]. Some bisbenzylisoquinoline alkaloids (tetrandrine and berbamine) inhibited neutrophil adhesion and locomotion [45, 46]. The alkaloid tetrandrine had also immunosuppressive effects and blocked T cell signaling [47, 48] and the isoquinoline alkaloid berberine was shown to block an inflammatory T cell response and the expression of costimulatory molecules by dendritic cells [49]. One of the mechanisms described to corroborate the inhibitory effect of such alkaloids on neutrophils adhesion was the antioxidant effect of substances like tetrandrine, which inhibited ROS formation [48]. This does not seem to be the case for warifteine, since this substance had no effect on the oxidative burst stimulated* in vitro* by PMA. Another mechanism that imparts anti-inflammatory effect to bis-benzylisoquinoline alkaloids is the inhibition of secretion of cytokines like IL-1, TNF-*α*, IL-6, and IL-8 [48, 50]. We did not test the production of all these cytokines in our system, but inhibition of cytokine production may not be the mechanisms through which warifteine would exert an anti-inflammatory response, since we did not observe a decrease in the secretion of TNF-*α* by neutrophils stimulated in the presence of warifteine (data not shown).

Neutrophil transmigration is the last step in the process of neutrophil response to an inflammatory stimulus* in vivo *[18] and this response was also inhibited by warifteine, as observed in transwell migration assays. Previous studies analyzing the effect of warifteine in B cell response have shown that this alkaloid decreased the activation-induced intracellular calcium increase [14]. Also, this alkaloid was shown to inhibit calcium channels and modified the intracellular Ca^2+^ stores in smooth muscle cells [11]. Therefore, the effect of warifteine in cell migration could be due to its effect in intracellular calcium levels in neutrophils. Even though we did not measure calcium levels in warifteine-treated cells, the alkaloid did not alter cytoskeleton movement in either PMA or fMLP-treated cells (data not shown). Other studies have characterized alkaloids that inhibit calcium signaling [51, 52] and have suggested that the addition of rolipram or dibutyryl cyclic AMP to neutrophil cultures would decrease calcium levels in fMLP-stimulated cells due to an enhancement of cyclic AMP-dependent calcium sequestration [53]. This may be occurring in warifteine-treated cultures, since we observed that warifteine indeed increased the levels of intracellular cAMP and that the blocking effect of warifteine in cell migration was in fact mimicked by* in vitro *treatment with a cAMP analog (DbcAMP) and by incubation with the adenyl cyclase activator forskolin.

During an infection, after migration to inflamed tissue, neutrophils would destroy local infecting microorganisms. A recently described microbicidal mechanism is the production of NETs [22]. We observed that warifteine inhibited NET formation in neutrophils. NET formation was shown to be dependent on ROS produced by the NADPH oxidase, elastase, hydrogen peroxide, and peptidyl arginine deiminase-4 (PAD4) through a mechanism poorly understood [54–57]. Our finding that fMLP-induced NET release was inhibited by warifteine despite no effect on the stimulation of oxidative burst by PMA suggested that additional steps exist between these two biological phenomena and this unidentified biochemical step was subjected to inhibition by warifteine. Although the physiological or pathological roles of NETs were unknown, these structures were identified in human atopic subjects [28]. In this milieu, NETs could protect airways from infections through their microbicidal properties, contribute to the airways damage and/or remodeling, and also modulate the immune response involved in asthma development [28, 58].

## 5. Conclusion

A hallmark feature of an inflammatory response is the infiltration of activated neutrophils and this response is altered by several anti-inflammatory drugs. Inhibition of neutrophil migration is a rational target for treating inflammation and drugs that inhibit this event would be an important option for the treatment of this disease alone or in association with other drugs. Our findings described here indicated that the alkaloid warifteine was a potent anti-inflammatory substance that had an effect on neutrophil migration through both a decrease in cell adhesion and migration. Warifteine also inhibited antimicrobial events like NET production. These characteristics impart to warifteine a potential use in the treatment of inflammatory diseases and pathological conditions associated with NETs production.
